# Novel Cellular Functions of Very Long Chain-Fatty Acids: Insight From ELOVL4 Mutations

**DOI:** 10.3389/fncel.2019.00428

**Published:** 2019-09-20

**Authors:** Ferenc Deák, Robert E. Anderson, Jennifer L. Fessler, David M. Sherry

**Affiliations:** ^1^Department of Geriatric Medicine, Reynolds Oklahoma Center on Aging, University of Oklahoma Health Sciences Center, Oklahoma City, OK, United States; ^2^Oklahoma Center for Neurosciences, University of Oklahoma Health Sciences Center, Oklahoma City, OK, United States; ^3^Harold Hamm Diabetes Center, University of Oklahoma Health Sciences Center, Oklahoma City, OK, United States; ^4^Dean McGee Eye Institute, University of Oklahoma Health Sciences Center, Oklahoma City, OK, United States; ^5^Department of Ophthalmology, University of Oklahoma Health Sciences Center, Oklahoma City, OK, United States; ^6^Department of Cell Biology, University of Oklahoma Health Sciences Center, Oklahoma City, OK, United States; ^7^Department of Pharmaceutical Sciences, University of Oklahoma Health Sciences Center, Oklahoma City, OK, United States

**Keywords:** very long chain-fatty acids, seizure, neurodegeneration, spinocerebellar ataxia, Stargardt’s-like macular dystrophy

## Abstract

Elongation of Very Long chain fatty acids-4 (ELOVL4) protein is a member of the ELOVL family of fatty acid elongases that is collectively responsible for catalyzing formation of long chain fatty acids. ELOVL4 is the only family member that catalyzes production of Very Long Chain Saturated Fatty Acids (VLC-SFA) and Very Long Chain Polyunsaturated Fatty Acids (VLC-PUFA) with chain lengths ≥28 carbons. ELOVL4 and its VLC-SFA and VLC-PUFA products are emerging as important regulators of synaptic signaling and neuronal survival in the central nervous system (CNS). Distinct sets of mutations in *ELOVL4* cause three different neurological diseases in humans. Heterozygous inheritance of one set of autosomal dominant *ELOVL4* mutations that leads to truncation of the ELOVL4 protein causes Stargardt-like macular dystrophy (STGD3), an aggressive juvenile-onset retinal degeneration. Heterozygous inheritance of a different set of autosomal dominant *ELOVL4* mutations that leads to a full-length protein with single amino acid substitutions causes spinocerebellar ataxia 34 (SCA34), a late-onset neurodegenerative disease characterized by gait ataxia and cerebellar atrophy. Homozygous inheritance of a different set of *ELOVL4* mutations causes a more severe disease with infantile onset characterized by seizures, spasticity, intellectual disability, ichthyosis, and premature death. ELOVL4 is expressed widely in the CNS and is found primarily in neurons. ELOVL4 is expressed in cell-specific patterns within different regions of the CNS that are likely to be related to disease symptoms. In the retina, ELOVL4 is expressed exclusively in photoreceptors and produces VLC-PUFA that are incorporated into phosphatidylcholine and enriched in the light sensitive membrane disks of the photoreceptor outer segments. VLC-PUFA are enzymatically converted into “elovanoid” compounds that appear to provide paracrine signals that promote photoreceptor and neuronal survival. In the brain, the main ELOVL4 products are VLC-SFA that are incorporated into sphingolipids and enriched in synaptic vesicles, where they regulate kinetics of presynaptic neurotransmitter release. Understanding the function of ELOVL4 and its VLC-SFA and VLC-PUFA products will advance our understanding of basic mechanisms in neural signaling and has potential for developing novel therapies for seizure and neurodegenerative diseases.

## Introduction

Lipids are critical biochemical components of the central nervous system (CNS) that are essential for proper CNS function. The lipid composition of the brain is unique and exceedingly diverse (Sastry, 1985; Bozek et al., 2015; Hopiavuori et al., 2017; Hopiavuori et al., 2018). Aberrant lipid composition, metabolism, and signaling in the CNS is associated with neuropsychiatric and neurodegenerative diseases. Aging also is known to alter lipid composition in the brain (see Sebastiao et al., 2013; Muller et al., 2015; Lauwers et al., 2016; Spassieva et al., 2016). In general, lipids are known as key participants in membrane structures and for their role in cell signaling (Bazinet and Laye, 2014; Sanchez-Alegria et al., 2018). Recently, lipids also have been suggested to serve as regulators of synaptic transmission (Marza et al., 2008; Brodde et al., 2012; Sebastiao et al., 2013; Carta et al., 2014; Lauwers et al., 2016), and a number of lipid metabolism enzymes have been localized to synaptic terminals where they would be positioned to provide local regulation of synaptic transmission (Cremona et al., 1999; Di Paolo et al., 2004; Rohrbough et al., 2004). However, these studies focused primarily on lipids with fatty acid chains of 16–22 carbons in length. Our understanding of the functions of fatty acids with longer chain lengths in the nervous system is more limited.

**FIGURE 1:**
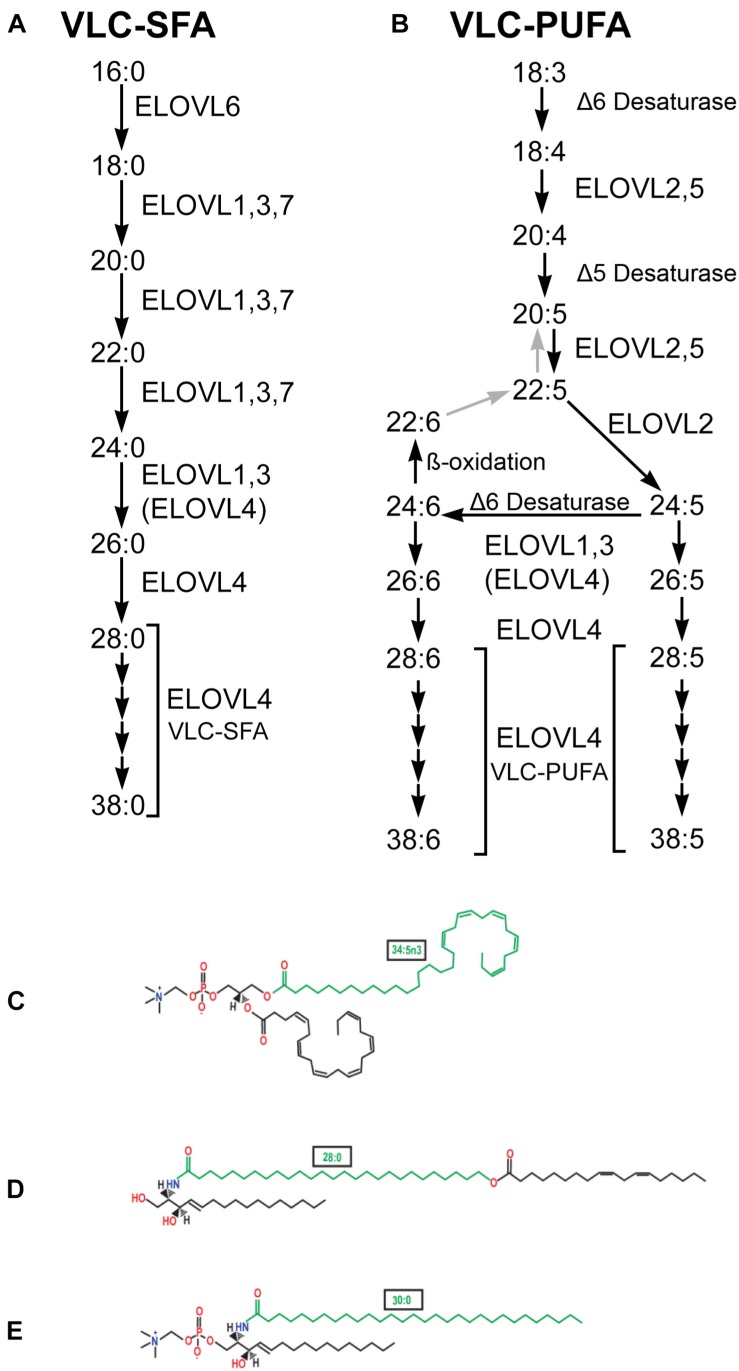
VLC-SFA and VLC-PUFA elongation pathways. **(A)** VLC-SFA biosynthesis pathway. Elongation steps are performed by ELOVL1-7. Although some ELOVL family members catalyze specific steps in VLC-SFA synthesis, others are multifunctional and may catalyze multiple steps. Elongation of C24 substrates also may be performed by ELOVL4 (ELOVL4). **(B)** VLC-PUFA biosynthesis pathway. Desaturation and elongation steps are performed by ELOVL1-5, Δ5 Desaturase (fatty acid desaturase-1, FADS1), and Δ6 desaturase (fatty acid desaturase-2, FADS2) as indicated. Although some ELOVL family members catalyze specific steps in VLC-PUFA synthesis, others are multifunctional and may catalyze multiple steps. Elongation of C24 substrates also may be performed by ELOVL4 (ELOVL4). **(C)** VLC-PUFA are incorporated into phosphatidylcholine in the retina. Example shown contains the VLC-PUFA, 34:5n3 (green), and the long chain-PUFA, 22:6n3 (DHA). **(D)** VLC-SFA are incorporated into ω-O-acylceramides in the skin. Example shown contains the VLC-SFA, 28:0 (green) ω-O-linked with 18:2n3. **(E)** VLC-SFA are incorporated into sphingolipids in the brain. Example shown shows sphingomyelin containing the VLC-SFA, 30:0 (green) (panels C–E from Hopiavuori et al., 2019, used with permission).

Recent studies have revealed novel functions for Very Long Chain-Fatty Acids (VLC-FA), defined by a chain length of 28 or more carbons, in neural signaling (Bennett et al., 2014a, b; Bhattacharjee et al., 2017; Jun et al., 2017; Hopiavuori et al., 2018). This review will discuss the seven known members of the Elongation of Very Long Chain-Fatty Acids (ELOVL) family of enzymes responsible for elongation of saturated and polyunsaturated fatty acids (Figure 1). In particular, we will focus on ELOVL4 and the role of its Very Long Chain-Saturated Fatty Acids (VLC-SFA) and Very Long Chain-PolyUnsaturated Fatty Acids (VLC-PUFA) products in neurological disease, synaptic transmission, and neuronal survival in the CNS. By convention, *ELOVL4* refers to the human gene, *Elovl4* to the non-human mammalian gene, and *elovl4* to non-mammalian gene. ELOVL4 refers to the protein in all species.

### The ELOVL Family of Fatty Acid Elongases

**FIGURE 2:**
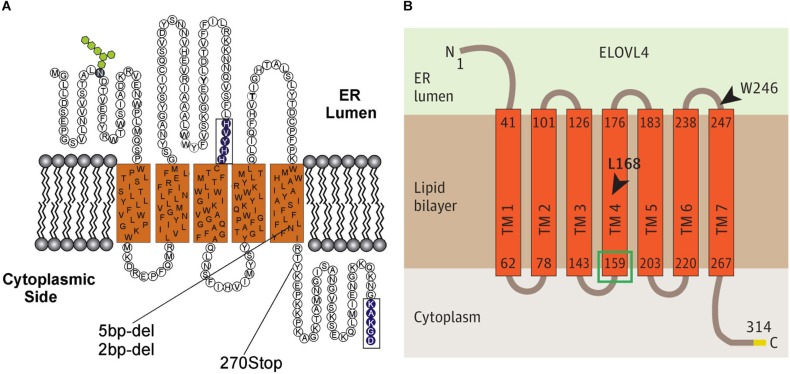
Predicted transmembrane topology for ELOVL4. **(A)** Predicted five transmembrane-spanning topology for ELOVL4 (Zhang et al., 2001; Molday and Zhang, 2010). **(B)** Predicted seven transmembrane-spanning topology for ELOVL4 (Ozaki et al., 2015; figures used with permission).

The ELOVL family of enzymes in mammals is comprised of seven members that all reside in the endoplasmic reticulum (ER) and are thought to form a multimeric complex (Okuda et al., 2010). Together, the ELOVL family is responsible for the elongation of saturated and unsaturated fatty acids (Guillou et al., 2010; Kihara, 2012; Yu et al., 2012). ELOVL4, specifically, is essential for the biosynthesis of VLC-SFA and VLC-PUFA (Agbaga et al., 2008). Each member of the ELOVL family is a multi-pass transmembrane protein containing a large ELO domain with a high degree of homology to a family of fatty acid elongases in yeast (Zhang et al., 2003), an N-linked glycosylation near the N-terminus, a catalytic histidine motif (HXXHH) that is essential for the elongase function (Logan et al., 2014), and a di-lysine ER-retention motif (KXKXX) located in the C-terminus domain that is required for proper localization to the ER (Logan et al., 2013). The initial topological model of ELOVL protein structure, based on ELOVL4, predicted five transmembrane domains (SOUSI model; Figure 2A; Zhang et al., 2001; Molday and Zhang, 2010). More recent modeling, using a variety of bioinformatics tools (MEMSAT-SVM, MEM-SAT3; ENSEMBLE, Phobius, and TMHMM2 models; Figure 2B), predicts seven transmembrane domains (Ozaki et al., 2015). In both models, the N-terminus and N-linked glycosylation site are located in the ER lumen, and the C-terminus ER-retention motif is located on the cytoplasmic side of the ER membrane. A key difference between the 5- and 7-membrane spanning topologies is the placement of the catalytic histidine motif. The five transmembrane spanning topology places this motif in or near the ER lumen close to the start of the third transmembrane domain. The seven transmembrane spanning topology places this motif near the beginning of the fourth transmembrane domain on the cytoplasmic side of the ER membrane. The precise structure of the ELOVL proteins remains unresolved as no crystal structures of any full-length ELOVL proteins are available to date.

**FIGURE 3:**
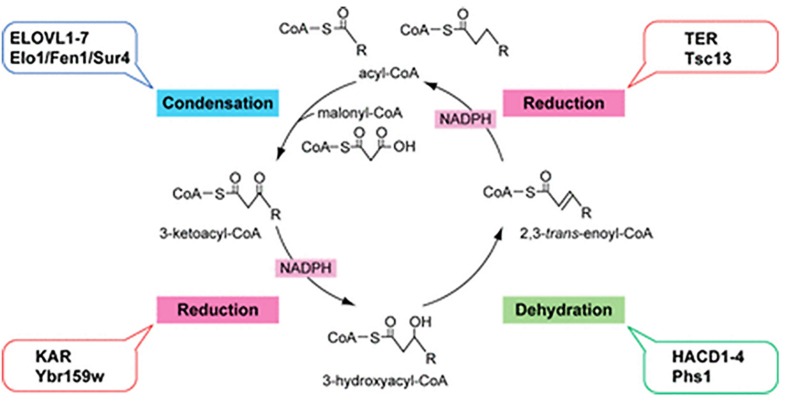
Fatty acid elongation by the ELOVL family of enzymes. Diagram of the four-step fatty acid elongation cycle indicating the mammalian (ALL CAPITALS) and the yeast enzymes involved in each step. The ELOVL family elongasaes catalyze the initial condensation reaction (Kihara, 2012; figure used with permission).

Fatty acid elongation occurs by cycling through a four step process (condensation, reduction, dehydration, and reduction), with two carbon atoms added through each cycle. Members of the ELOVL family catalyze the first step, a condensation reaction between a fatty acyl-CoA and malonyl-CoA (Pereira et al., 2004; Jakobsson et al., 2006). The second and fourth reduction steps are catalyzed by 3-ketoacyl-CoA and trans-2,3-enoyl-CoA reductases (KAR and TER), respectively, the third dehydration step is catalyzed by 3-hydroxyacyl-CoA dehydratases (HACD1-4) (Figure 3; Moon and Horton, 2003; Konishi et al., 2010). The ELOVL family proteins are thought to form hetero-oligomeric complexes in the ER (Okuda et al., 2010).

Complexing of ELOVL proteins is important to their function (Okuda et al., 2010; Logan et al., 2014). ELOVL4 is known to homodimerize, with dimerization of WT and STDG3 mutant ELOVL4 causing mislocalization of the complex away from the ER (Grayson and Molday, 2005; Molday and Zhang, 2010) and exerting a dominant negative effect on enzyme function (Logan et al., 2014). In addition, ELOVL4 can hetero-oligomerize with other ELOVL family members and also can complex with other enzymes associated with VLC-FA elongation (Okuda et al., 2010). Furthermore, STDG3 mutants of ELOVL4 interact with other ELOVL family elongases and other VLC-FA-associated enzymes more strongly that WT ELOVL4, suggesting that mutant forms of ELOVL4 also might affect synthesis of other fatty acid species in addition to VLC-FA (Okuda et al., 2010).

The ELOVL family of elongases is involved in elongation of many different lipid species. Importantly, some ELOVL family elongases show distinct substrate selectivity and mediate very specific elongation reactions, while other members of the family show broader substrate selectivity and can show functional redundancy (Guillou et al., 2010; Kihara, 2012; Yu et al., 2012). Thus, the ELOVL family mediates a wide range of fatty acid elongation reactions leading to a diverse array of PUFA and SFA products.

ELOVL4 mediates elongation of long chain PUFA and SFA to form VLC-PUFA and VLC-SFA of 28 carbon chain length, respectively (Figure 1). ELOVL4 can then further elongate VLC-PUFA and VLC-SFA of 28 carbon chain length to produce VLC-PUFA and VLC-SFA species with chain lengths up to 38 carbons (Agbaga et al., 2008, 2010b). With respect to formation of VLC-PUFA, both 20:5n3 (eicosapentaenoic acid, EPA) and 22:5n3 (docosapentaenoic acid, DPA) precursors support downstream synthesis of VLC-PUFA (Agbaga et al., 2008). EPA is preferred as a substrate for elongation to VLC-PUFA over 20:4n6 (arachidonic acid, AA) and 22:6n3 (docosahexaenoic acid, DHA) (Yu et al., 2012). Importantly, ELOVL4 does not elongate shorter chain polyunsaturated fatty acids to DHA (Agbaga et al., 2010a). Formation of VLC-SFA is accomplished by ELOVL4-mediated elongation of 26:0–28:0, which can then be elongated further by ELOVL4 to produce VLC-SFA with longer carbon chains (Guillou et al., 2010; Kihara, 2012). The major VLC-SFA products of ELOVL4 in the brain are 28:0 and 30:0 (Hopiavuori et al., 2018).

The extreme length of the VLC-SFA and VLC-PUFA confers unique properties to the complex lipids and membranes into which they are incorporated. The very long, linear carbon chain of VLC-SFA confers a high melting temperature and would increase membrane stiffness through Van der Waals interactions between adjacent alkyl chains. Furthermore, the linear structure of VLC-SFA would be of sufficient length to span across the leaflets of the lipid bilayer, further affecting membrane properties (Hopiavuori et al., 2018, discussed further below). In contrast, VLC-PUFA, due to their length and multiple methylene interrupted *cis* double bonds, lead to locally disordered phospholipid packing in the membrane, increased fluidity, and potentially affect membrane curvature (Antonny et al., 2015; Lauwers et al., 2016).

The VLC-PUFA and VLC-SFA products of ELOVL4 are generated in a tissue-specific manner and are incorporated into more complex lipids in a tissue-specific manner. In the skin, the major products of ELOVL4 are VLC-SFA, as shown by the fact that epidermal fatty acids longer than C26 are virtually absent in newborn mice lacking a functional Elovl4 protein (Cameron et al., 2007). The molecular species of skin ceramides and glucosylceramides (GlcCer) contain VLC-SFA in non-hydroxy, α-hydroxy, and ω-hydroxy forms, the latter occurring in non-esterified and esterified forms (mostly with 18:2) (Figure 1). Strikingly, the skin of Elovl4^–/–^ mice is devoid of the epidermal unique ω-O-acylceramides (> C30) (McMahon et al., 2007; Vasireddy et al., 2007). Part of the ω-hydroxy-Ceramide and GlcCer species are esterified to specific skin proteins (Amen et al., 2013), and are critical to establishing the extremely hydrophobic extracellular lipid lamellae of the stratum corneum that serves as the water barrier for the skin. Similarly, ELOVL4 in the Meibomian gland also generates VLC-SFA that are incorporated into ω-O-acylceramides, which are essential components of the lipid layer that covers the aqueous tear film and prevents evaporation (McMahon et al., 2014). In the testes, ELOVL4 produces VLC-PUFA (Santiago Valtierra et al., 2018), which are incorporated into sphingolipid products via ceramide synthase 3 (CerS3) (Rabionet et al., 2008). Conditional deletion of CerS3 leads to the absence of virtually all of sphingolipid products that contain VLC-PUFA, and infertility due to enhanced apoptosis during meiosis and spermatogenic arrest (Rabionet et al., 2015). VLC-PUFA are likely to be important to human fertility, as decreased VLC-PUFA levels in sperm are associated with decreased sperm quantity and quality (Craig et al., 2019).

In the CNS, ELOVL4 produces both VLC-SFA and VLC-PUFA in a region-specific manner. In the brain, the main ELOVL4 products are VLC-SFA, which are incorporated in sphingolipids that are enriched in synaptic vesicles and regulate presynaptic release (Hopiavuori et al., 2018). In retina, the main ELOVL4 products are VLC-PUFA (Agbaga et al., 2008, 2010b), which are incorporated into phosphatidylcholine and enriched in the light sensitive photoreceptor outer segments (Aveldano, 1987). VLC-PUFA are critical to photoreceptor health and survival (Agbaga et al., 2008, 2010b; Bennett et al., 2014a, b; Bhattacharjee et al., 2017; Jun et al., 2017). VLC-PUFA have been reported previously in the brain (Poulos et al., 1988; Robinson et al., 1990). Although VLC-PUFA are present in the brain only in trace amounts in health, phosphatidylcholine species containing VLC-PUFA were reported previously in the brain of newborn children affected with Zellweger disease (Poulos et al., 1988), a rare peroxisomal biogenesis disorder in which in the oxidation of VLC-FA is impaired. Such PC species also were detected in the brain of healthy newborn rats in small amounts, decreasing substantially from postnatal days 1 to 16 and being virtually absent at 60 days (Robinson et al., 1990). Recent exhaustive lipidomic studies of healthy wildtype adult mouse brain did not detect these species (Hopiavuori et al., 2017).

### The Function of the Mammalian ELOVL Elongase Family in Health and Disease

The mammalian ELOVL family of elongases has a number of important functions in the body and, as a group, are particularly important to CNS function, the epidermal water barrier, systemic metabolic functions, and also are likely to be important to fertility. Mutations affecting *ELOVL1*, *-4*, *-5*, and *-7* are associated with neurological disease. Importantly, the diseases caused by these mutations have some shared characteristics that suggest fatty acid elongation in general is particularly critical to the health of the CNS. No disease causing mutations in *ELOVL2*, *-3*, or *-6* have been reported to date. The synthetic activities and functions of each ELOVL family member are summarized below.

#### ELOVL1

ELOVL1 elongates SFA with chain lengths of 18–24 carbons and shows functional redundancy with ELOVL3 and ELOVL7 (Figure 1; Guillou et al., 2010; Ohno et al., 2010; Kihara, 2012). Deletion of *Elovl1* in mice causes disruption of the lamellae in the stratum corneum of the skin and disrupts the water barrier of the skin leading to perinatal lethality (Sassa et al., 2013). *Elovl1* mutant mice also show deficiency of VLC-SFA and VLC-mono-unsaturated fatty acids in the tear film (Sassa et al., 2018). *ELOVL1* is expressed at moderate levels in the brain (Tvrdik et al., 2000). An autosomal dominant mutation in human *ELOVL1* has recently been reported that produces ichthyosis, hypomyelination, spastic paraplegia, partial deafness, and optic atrophy (Mueller et al., 2019). This syndrome shares some symptoms with the neuro-ichtyotic syndrome caused by autosomal recessive mutations in *ELOVL4* (Aldahmesh et al., 2011; Mir et al., 2014; discussed below).

#### ELOVL2

ELOVL2 elongates PUFA with 20–22 carbon and SFA with 18–20 carbon chains (Figure 1; Guillou et al., 2010; Zadravec et al., 2011; Kihara, 2012)and has low expression in the brain (Tvrdik et al., 2000). Knockout studies in mice indicate that *Elovl2* is essential for normal lipid homeostasis (Pauter et al., 2014). No human diseases arising from *ELOVL2* mutations have been reported to date, although polymorphisms with genetic linkage to autism have been reported recently (Sun et al., 2018). Interestingly, human epigenetic screening indicates that the *ELOVL2* gene shows a progressive increase in methylation that begins at an early stage of life and is a promising biomarker for aging in all tissues (Garagnani et al., 2012; Gopalan et al., 2017; Slieker et al., 2018; Jung et al., 2019) that can be used for forensic age determination (Spolnicka et al., 2018).

#### ELOVL3

ELOVL3 elongates SFA with chain lengths of 18–24 carbons and can be functionally redundant with ELOVL1 and ELOVL7 (Figure 1; Westerberg et al., 2004; Guillou et al., 2010; Kihara, 2012). Knockout of *Elovl3* in mice disrupts the water barrier of the skin and causes hyperplasia of the sebaceous glands and hair loss (Westerberg et al., 2004; Kihara, 2012). No linkage to CNS disease in humans has been reported for ELOVL3.

#### ELOVL4

ELOVL4 elongates long-chain PUFA and long-chain SFA of 24 carbon length to VLC-PUFA and VLC-SFA (≥26 carbons; Figure 1; Agbaga et al., 2008, 2010b; Guillou et al., 2010; Kihara, 2012). ELOVL4 also can further elongate VLC-PUFA and VLC-SFA up to 38 carbons in length. No other ELOVL family member performs this function. Thus, there is no compensation for mutations that compromise the ability of ELOVL4 to synthesize VLC-PUFA or VLC-SFA. Mutations in *ELOVL4* cause three different human diseases with tissue-specific characteristics: Stargardt-like macular dystrophy (STGD3), spinocerebellar ataxia 34 (SCA34), and a neuro-ichthyotic syndrome. All of these diseases have profound effects on the CNS and are discussed in more detail in several sections below.

#### ELOVL5

ELOVL5 mediates elongation of long chain-PUFA and long chain-SFA between 18 and 22 carbons in length (Figure 1; Leonard et al., 2002; Guillou et al., 2010; Kihara, 2012). Two different mutations in *ELOVL5* cause spinocerebellar ataxia 38 (SCA38) in humans, which is characterized by gait ataxia, nystagmus, anosmia, and cerebellar atrophy (Di Gregorio et al., 2014). In the cerebellum, ELOVL5 is highly expressed in Purkinje cells, which provide the sole output from the cerebellar cortex, and in some cells of unidentified type located in the granule cell layer (Di Gregorio et al., 2014; Hoxha et al., 2017).

#### ELOVL6

ELOVL6 has been suggested to mediate the first, rate-limiting step in fatty acid elongation of saturated and unsaturated/polyunsaturated fatty acids with chain length of 16 carbons, and is expressed at high levels in the liver, adipose tissue, and brain (Figure 1; Moon et al., 2001; Guillou et al., 2010; Kihara, 2012; Moon et al., 2014). *Elovl6* knockout mice are insulin resistant and develop obesity and hepatosteatosis when fed a high fat diet (Matsuzaka et al., 2007), indicating that ELOVL6 is important to normal metabolic regulation. The activity of ELOVL6 can also regulate thermogenic activity in brown fat adipocytes (Tan et al., 2015) and chondrocyte growth (Kikuchi et al., 2016). No direct causal linkage of *ELOVL6* mutations to human disease has been established.

#### ELOVL7

ELOVL7 elongates SFA with chain lengths of 18–22 carbons and shows some functional redundancy with ELOVL1 and ELOVL3 (Guillou et al., 2010; Naganuma et al., 2011; Kihara, 2012). No direct causal relationship between ELOVL7 and human disease has been established. However, association of single nucleotide polymorphisms in *ELOVL7* with early onset Parkinson’s disease has been reported (Li et al., 2018). A case of apparent mitochondrial encephalomyopathy arising from homozygous deletion of the contiguous *NDUFAF2*, *ERCC8*, and *ELOVL7* genes on chromosome 5 has been reported (Janssen et al., 2009). No changes in fatty acid synthesis were found, suggesting that deletion of *ELOVL7* is unlikely to be the root cause of the disease.

### Non-mammalian ELOVL Family Fatty Acid Elongases

ELOVL family fatty acid elongases in fish also have been of particular interest because of their function as source of polyunsaturated fatty acids. As indicated above, ELOVL family elongases are highly conserved across eukaryotes, and homologs of mammalian ELOVL2, ELOVL4, and ELOVL5 have been identified and characterized functionally in several species of teleost fish, including zebrafish, Salmon, cobia, and Chu’s croaker (Hastings et al., 2004; Morais et al., 2009; Monroig et al., 2010, 2011; Carmona-Antonanzas et al., 2011; Lin et al., 2018). For more detailed discussion of ELOVL gene evolution (see Castro et al., 2016). A single isoform of ELOVL2 has been identified in fish (Morais et al., 2009). ELOVL5 is expressed in a single isoform in some fish species (i.e., Chu’s croaker, Lin et al., 2018), while other species express two functionally similar isoforms (ELOVL5a and b) (i.e., Atlantic salmon, Hastings et al., 2004; Morais et al., 2009). ELOVL2 and ELOVL5 are functionally redundant with one another and serve functions similar to their mammalian homologs: ELOVL2 elongates C20 and C22 PUFA to C24 PUFA; ELOVL5a and 5b elongate C18 and C20 PUFA to C22 PUFA (Carmona-Antonanzas et al., 2011).

Similar to mammals, ELOVL4 in fish is highly expressed in brain, retina, and gonads and is essential for formation of VLC-SFA and VLC-PUFA (Monroig et al., 2010, 2011; Carmona-Antonanzas et al., 2011). However, ELOVL4 in fish can be expressed in a single isoform (i.e., Atlantic salmon, cobia Carmona-Antonanzas et al., 2011; Monroig et al., 2011) or two isoforms (ELOVL4a and b) that are encoded by separate genes (i.e., zebrafish, Monroig et al., 2010). ELOVL4a and ELOVL4b show very distinct substrate specificities: ELOVL4b readily elongates both VLC-SFA and VLC-PUFA, but ELOVL4a elongates only VLC-SFA (Monroig et al., 2010). In fish that express only a single ELOVL4 isoform, ELOVL4 shows activity similar to zebrafish ELOVL4b and elongates both VLC-SFA and VC-PUFA (Monroig et al., 2010; Carmona-Antonanzas et al., 2011). Fish ELOVL4 also can elongate C20 and C22 substrates, making it functionally redundant with ELOVL2 and to a lesser extent ELOVL5 (Carmona-Antonanzas et al., 2011), in contrast to mammalian ELOVL4. Importantly, this broad substrate selectivity could allow fish ELOVL2 and ELOVL4 to participate in the synthesis of DHA, as these enzymes can catalyze synthesis of 24:5n-3 from 22:5n-3, allowing for subsequent desaturation and β-oxidation to form DHA (Hastings et al., 2004; Monroig et al., 2010, 2011; Carmona-Antonanzas et al., 2011).

### Distribution of the ELOVL Family in the CNS

Several members of the ELOVL family are expressed in the CNS (ELOVL1, -3, -4, -5, and -6), but their expression levels differ across brain regions (Tvrdik et al., 2000; Lein et al., 2007; Hoxha et al., 2017; Sherry et al., 2017). ELOVL2 and ELOVL7 are expressed at very low levels in mammalian brain, if at all (Tvrdik et al., 2000; Lein et al., 2007), although ELOVL2 is expressed highly in the brain of non-mammals (Oboh et al., 2016). The best characterized ELOVL family members in the mammalian brain are ELOVL4 and ELOVL5 (Hoxha et al., 2017; Sherry et al., 2017; Hopiavuori et al., 2018). Mutations in either *ELOVL4* or *ELOVL5* cause neurological disease in humans (see below and Table 1). Interestingly, zebrafish ELOVL4a, which catalyzes formation of VLC-SFA only, is highly expressed in brain, and ELOVL4b, which catalyzes production of VLC-PUFA as well as VLC-SFA, is highly expressed in retina (Monroig et al., 2010; Carmona-Antonanzas et al., 2011). This distribution of ELOVL4 isoforms would result in similar patterns of VLC-SFA and VLC-PUFA production in the brains and retinae of teleost fish and mammals. ELOVL5 also is expressed at high levels in the fish brain (Kohlhardt, 1989), similar to mammalian brain.

**TABLE 1:** Summary of human disease-causing *ELOVL4* mutations.

**Human disease**	**Inheritance**	**Genomic mutation**	**Protein mutation/structure/function**	**Retinal symptoms**	**Other CNS symptoms**	**Skin symptoms**	**Onset/Progression**	**References**
Wildtype ELOVL4.	Homozygous	Wildtype (no mutation)	Wildtype. Intrinsic endoplasmic reticulum membrane protein. 314 AA length	–	–	–	–	Edwards et al., 2001; Zhang et al., 2001
Stargardt’s-like macular dystrophy (STGD3)	Autosomal dominant	790–794 del AACTT “5 bp deletion”	Exon 6, N264Lfs10X	Macular degeneration	None reported	None	Juvenile onset. Rapid progression.	Edwards et al., 2001; Zhang et al., 2001
Stargardt’s-like macular dystrophy (STGD3)	Autosomal dominant	789 del T *plus* 794 del T “2 bp deletion”	Exon 6, N264Tfs9X	Macular degeneration	None reported	None	Juvenile onset. Rapid progression.	Bernstein et al., 2001
Stargardt’s-like macular dystrophy (STGD3)	Autosomal dominant	c.810C > G	Exon 6, Y270X	Macular degeneration	None reported	None	Juvenile onset. Rapid progression.	Maugeri et al., 2004
Stargardt’s-like macular dystrophy (STGD3)	Autosomal dominant	c.814G > C	Exon 6, E272Q	Macular degeneration	None reported	None reported	Juvenile onset. Rapid progression.	Bardak et al., 2016
Stargardt’s-like macular dystrophy (STGD3)	Autosomal dominant	c.895A > G	Exon 6, M299V	Macular degeneration	None reported	None reported	Juvenile onset. Rapid progression.	Bardak et al., 2016
Stargardt’s-like macular dystrophy (STGD3)	Autosomal dominant	c.-90 G > C	Promoter, rs62407622	Macular degeneration	None reported	None reported	Juvenile onset. Rapid progression.	Donato et al., 2018
Stargardt’s-like macular dystrophy (STGD3)	Autosomal dominant	c.-236 C > T	Promoter, rs240307	Macular degeneration	None reported	None reported	Juvenile onset. Rapid progression.	Donato et al., 2018
Spinocerebellar ataxia (SCA34)	Autosomal dominant	c.504G > C	Exon 4, L168F Full-length protein	Not reported	Gait and limb ataxia. Dysarthria. Nystagmus and eye movement deficits. Abnormal tendon reflexes. No autonomic disturbance. Cerebellar and pontine atrophy.	Erythrokerato-dermia Variabilis	Avg. onset: 51 years of age. Onset in 4th to 5th decade. Slow progression.	Cadieux-Dion et al., 2014
Spinocerebellar ataxia (SCA34)	Autosomal dominant	c.736T > G	Exon 4, W246G Full-length protein	Normal	Gait and limb ataxia. Dysarthria. Nystagmus and eye movement deficits. Abnormal tendon reflexes. Babinski reflex may be present. Autonomic disturbance sometimes present. Cerebellar and pontine atrophy.	None	Avg. onset: 34 years of age (range: 2nd–6th decade). Slow progression.	Ozaki et al., 2015
Spinocerebellar ataxia (SCA34)	Autosomal dominant	c.539A > C	Exon 4, Q180P Full length protein	None reported	Gait ataxia more pronounced than limb ataxia. Dysarthria. Nystagmus and eye movement deficits. Moderate cerebellar and pontine atrophy.	Erythrokerato-dermia Variabilis	Onset in middle 20 s (1 case known). Progression unclear at present	Bourassa et al., 2015
Spinocerebellar ataxia (SCA34)	Autosomal dominant	c698C > T	Exon 4, T233M Full length protein	None reported	Gait ataxia (no limb ataxia). No dysarthria. No nystagmus, but abnormal eye movements present. Hyporeflexia. Mild cerebellar and pontine atrophy.	Erythrokerato-dermia Variabilis	15 years of age (1 case known). Slow progression	Bourque et al., 2018
ELOVL4 Neuro-ichthyotic syndrome	Homozygous recessive	c.689delT	Exon 6, I230MfsX22	Normal fundus and flicker ERG	Seizures, intellectual disability, spastic quadriplegia	Ichthyosis	Infancy (Gestation?) Developmental delay	Aldahmesh et al., 2011
ELOVL4 Neuro-ichthyotic syndrome	Homozygous recessive	c.646C > T	Exon 5, R216X	Not reported	Seizures, intellectual disability, spastic quadriplegia	Ichthyosis	Infancy (Gestation?) Developmental delay	Aldahmesh et al., 2011
ELOVL4 Neuro-ichthyotic syndrome	Homozygous recessive	c.78C > G	Exon 1, Y26X	Not reported	Seizures, intellectual disability, spastic quadriplegia	Ichthyosis	Infancy (Gestation?) Developmental delay	Mir et al., 2014

#### ELOVL4 Distribution in the Brain

**FIGURE 4:**
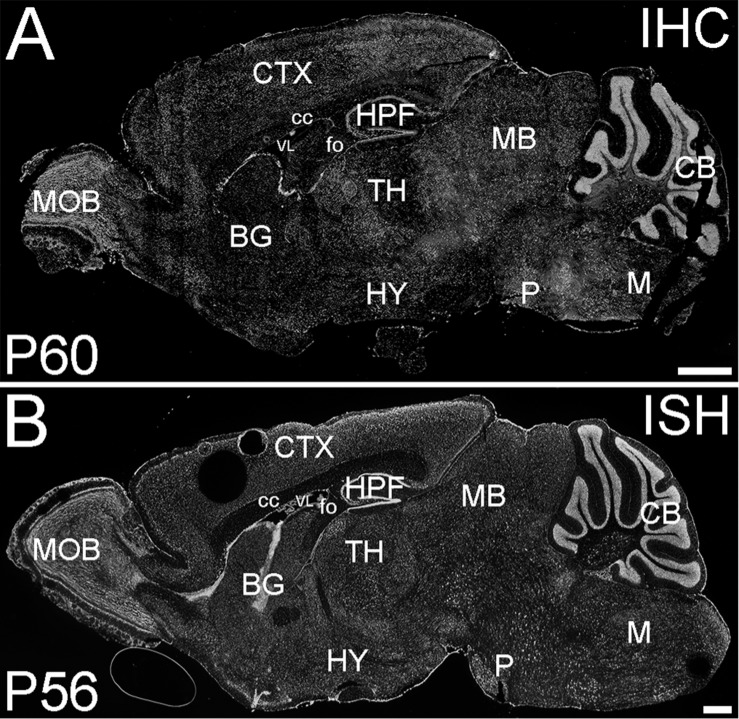
ELOVL4 protein and mRNA distribution in the adult mouse brain. **(A)** Immunofluorescent labeling for ELOVL4 in the postnatal day 60 mouse brain is widely distributed, but shows cell-specific distribution in different regions of the brain. **(B)** *In situ* hybridization (ISH) for *Elovl4* mRNA in the mouse brain at P56 shows distribution similar to that of ELOVL4 protein. (Image from: Allen Institute for Brain Science Allen Mouse Brain Atlas for the P56 mouse brain, image number 69059903_134; http://mouse.brain-map. org). CB, cerebellum; BG, basal ganglia; CTX, cerebral cortex; HPF, hippocampal formation; HY, hypothalamus; M, medulla; MB, midbrain; MOB, main olfactory bulb; P, pons; cc, corpus callosum; fo, fornix; VL, lateral ventricle. Scale bars = 1 mm (figure from Sherry et al., 2017). Used with open access under the Creative Commons attribution license CC-BY, version 4.0; http://creativecommons.org/licenses/by/4.0/).

ELOVL4 is the most highly expressed and widely distributed member of the ELOVL family in the brain as shown by immunolabeling and *in situ* hybridization (see Figure 4). ELOVL4 expression varies in a region- and cell type-specific manner (Lein et al., 2007; Sherry et al., 2017; Hopiavuori et al., 2018) which is likely to be related to the dysfunctions observed in diseases arising from *Elovl4* mutations. Expression of ELOVL4 is especially prominent in the olfactory bulb, hippocampus, cerebral cortex, thalamus, and cerebellum, although most other brain regions also show substantial levels of ELOVL4. An exception to this pattern is the basal ganglia, which show very little ELOVL4 expression (Sherry et al., 2017). At the cellular level, ELOVL4 expression is primarily neuronal, although small ELOVL4-positive cells have been observed in brain white matter suggesting potential expression in oligodendrocytes (Sherry et al., 2017). Among neurons, ELOVL4 expression is present in glutamatergic as well as GABAergic neurons (Sherry et al., 2017), and also may occur in neurons that utilize other neurotransmitters.

**FIGURE 5:**
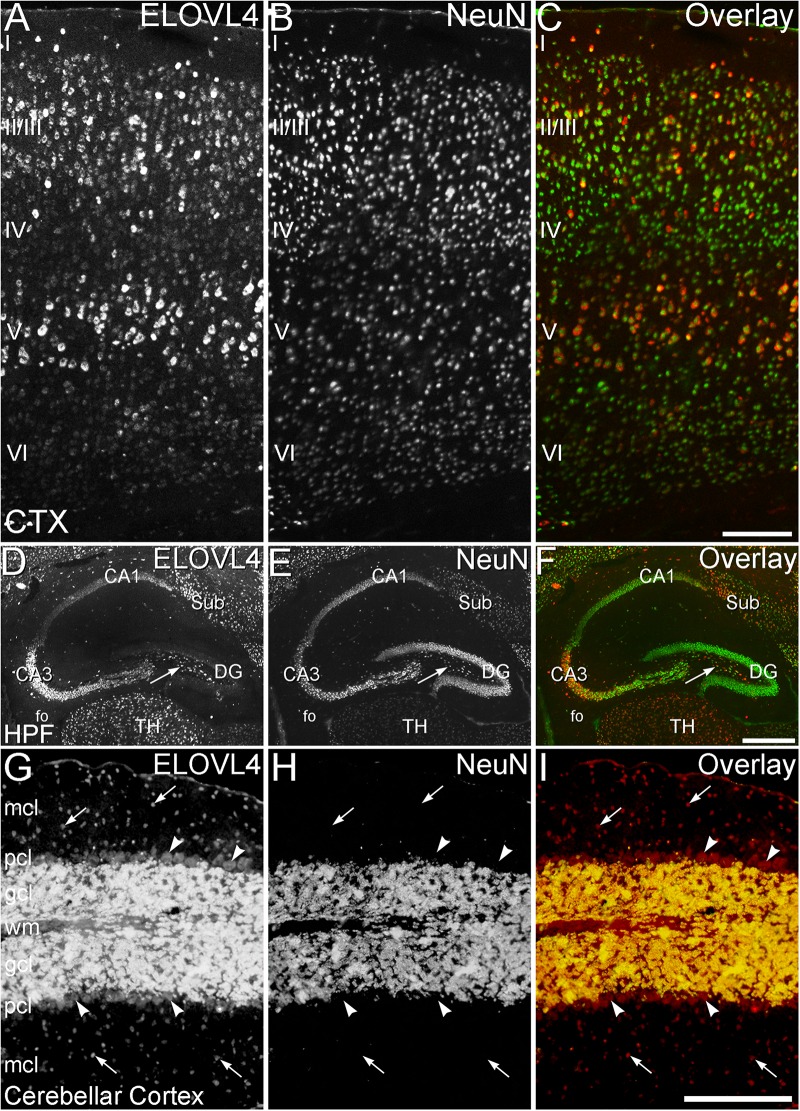
Cell-specific expression of ELOVL4 in isocortex, hippocampus and cerebellum. **(A–C)** Cerebral Cortex (CTX). **(A)** Labeling for ELOVL4 is present in all layers of the cerebral cortex (CTX). Cells in the pyramidal layers (II/III and V) are most prominently labeled, but ELOVL4-positive cells also are present in the molecular layer (I), layer 4 (IV) and layer 6 (VI). **(B)** Labeling for the neuronal marker, NeuN. **(C)** Overlay of panels A and B shows close correspondence of ELOVL4 (red) and NeuN labeling (green). **(D–F)** Hippocampal formation (HPF). **(D)** Labeling for ELOVL4 is present in the cellular layers of the HPF, including the Cornu Ammonis, with field 1 (CA1) showing less prominent labeling than field 3 (CA3). Prominent ELOVL4 labeling also is present in the subiculum (sub) and interneurons in the polymorph layer (arrow). Cells along the inner margin of the dentate gyrus (DG) show moderate ELOVL4 labeling, but most dentate granule cells show little ELOVL4 labeling. **(E)** Labeling for the neuronal marker, NeuN. **(F).** Overlay of **(D,E)** shows close correspondence of ELOVL4 (red) and NeuN (green) labeling. TH, Thalamus. **(G–I)** Cerebellar cortex. **(G)** Cross section through a cerebellar folium showing ELOVL4 expression in the cerebellar cortex. Neurons (arrows) in the molecular cell layer (mcl) show strong ELOVL4 labeling, but the Purkinje cells (arrowheads) in the Purkinje cell layer (pcl) show only moderate levels of ELOVL4 labeling. The densely packed cells of the granule cell layer (gcl) show very intense labeling. **(H)** Labeling for the neuronal marker, NeuN, strongly labels neurons in the gcl, but not Purkinje cells, as appropriate. **(I)** Overlay of **(G,H)** shows close correspondence of intense ELOVL4 (red) and NeuN (green) labeling resulting in an orange color in the gcl. wm, white matter of the arbor vitae. Scale bars = 200 μm for **(A–C,G–I)**; 500 μm for **(D–F)** (figure from Sherry et al., 2017; used with open access under the Creative Commons attribution license CC-BY, version 4.0; http://creativecommons.org/licenses/by/4.0/).

Within a region, ELOVL4 expression is cell-specific (Figure 5). In the retina, ELOVL4 is expressed exclusively by photoreceptor cells (Agbaga et al., 2008), consistent with the photoreceptor degeneration associated with ELOVL4 mutations that cause Stargardt’s-like macular dystrophy (STGD3) (Bernstein et al., 2001; Edwards et al., 2001; Zhang et al., 2001; Maugeri et al., 2004; Bardak et al., 2016; Donato et al., 2018). In the cerebellum, ELOVL4 levels are extremely high in granule cells, moderate in basket and stellate cells, and low in Purkinje cells (Sherry et al., 2017). These cell-specific differences in ELOVL4 expression may be related to the symptoms and progression of spinocerebellar ataxia-34 (SCA34), which is caused by human *ELOVL4* mutations (Cadieux-Dion et al., 2014; Bourassa et al., 2015; Ozaki et al., 2015; Bourque et al., 2018). In the hippocampus, the highest levels of ELOVL4 in neurons in the CA3 and CA4 regions, with low expression in CA1 and dentate gyrus (Sherry et al., 2017; Hopiavuori et al., 2018), consistent with the severe, spontaneous epileptiform bursting and seizure activity observed in mice homozygous for the 5bp deletion STGD3 mutant *ELOVL4* alleles (Hopiavuori et al., 2018). This phenotype is also consistent with the seizure activity reported in the recessive human *ELOVL4* neuro-ichthyotic syndrome (Aldahmesh et al., 2011; Mir et al., 2014).

Expression of ELOVL4 in the brain is developmentally regulated, with *Elovl4* mRNA expression levels peaking around the time of birth and then steadily falling as the brain matures, reaching a steady state level by about postnatal day 30 in mice (Mandal et al., 2004). Immunolabeling studies of the developing mouse brain between embryonic day 18 (E18) and postnatal day 60 (P60) show that ELOVL4 is very highly expressed in regions such as the subventricular zone, the dentate gyrus of the hippocampus, and the internal and external granular layers of the cerebellum during periods of neurogenesis (Sherry et al., 2017). ELOVL4 expression in these regions declines as neurogenesis declines, suggesting some role for ELOVL4 and its VLC-FA products in neurogenesis.

#### ELOVL5 Distribution in the Brain

ELOVL5 distribution has been characterized in the cerebellum, using immunolabeling of wildtype mouse cerebellum (Di Gregorio et al., 2014) and β-galactosidase reporter labeling in transgenic *Elovl5* knockout mice (Hoxha et al., 2017). ELOVL5 is expressed in cerebellar Purkinje cells, stellate and basket cells in the molecular layer, and at lower levels in a sparse, unidentified cell population in the granule cell layer of the cerebellar cortex and in cells in the deep cerebellar nuclei (Di Gregorio et al., 2014; Hoxha et al., 2017). Two different missense mutations in *ELOVL5* (c.214C > G, p.Leu72Val and c.689G > T, p.Gly230Val) cause SCA38, which is characterized by gait ataxia, dysarthria, abnormal eye movements, and cerebellar atrophy with onset in the third or fourth decade (Di Gregorio et al., 2014). ELOVL5 expression also was noted in small cells located in the white matter, most likely representing myelin-producing oligodendrocytes (Hoxha et al., 2017). Elsewhere in the brain, ELOVL5 is expressed by the mitral cells of the olfactory bulb, consistent with the anosmia (loss of the sense of smell) reported in *Elovl5* knockout mice, and in various sites in the brainstem (Hoxha et al., 2017). ELOVL5 also is expressed in other regions of the brain, but this has not been characterized in detail.

Thus, ELOVL4 and ELOVL5, and possibly other ELOVL enzyme family members, are expressed in a region- and cell-specific manner in the CNS, which would lead to cell-specific profiles of fatty acid synthesis and incorporation into complex lipids (Agbaga et al., 2018). This suggests that the effects of mutations on the enzymatic activity of ELOVL4, ELOVL5, and other lipid metabolizing enzymes are likely to be key determinants of the neurological symptoms associated with specific mutations in these enzymes. Developing a better understanding of the lipid profiles of specific cell types in the CNS will be important to advancing our mechanistic understanding of the role of VLC-FA in the healthy CNS and in neurological disease.

### Diseases Associated With ELOVL4

**FIGURE 6:**
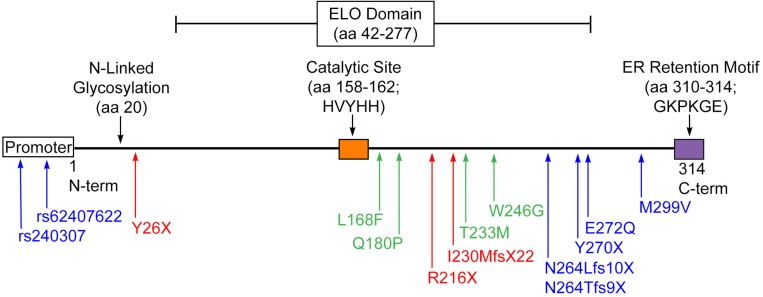
Disease-causing mutations in ELOVL4. STGD3-causing mutations (blue) result in early truncation of ELOVL4 and the loss of the C-terminal ER retention motif or, in the case of the promoter mutations, severely suppress *ELOVL4* expression. SCA34-causing mutations (green) result in amino acid substitutions but still produce a full-length protein. ELOVL4 Neuro-ichthyosis-causing mutations (red) cause very early termination of the protein that would truncate substantial portions of transmembrane domains in either the 5-transmembrane or 7-transmembrane models of ELOVL4 topology in addition to deleting the ER retention motif.

Several disease-causing mutations in the human *ELOVL4* gene have been identified to date (Bernstein et al., 2001; Edwards et al., 2001; Zhang et al., 2001; Maugeri et al., 2004; Aldahmesh et al., 2011; Cadieux-Dion et al., 2014; Mir et al., 2014; Bourassa et al., 2015; Ozaki et al., 2015; Bardak et al., 2016; Bourque et al., 2018; Donato et al., 2018; Table 1 and Figure 6). Mutations in *ELOVL4* cause three distinct neurodegenerative diseases that depend on the specific mutation and its pattern of inheritance: Stargardt-like macular dystrophy (STGD3), spinocerebellar ataxia-34 (SCA34) with or without erythrokeratodermia variabilis (EKV), and a severe neuro-ichthyotic syndrome. Each disease is discussed below.

#### STGD3

Six different heterozygous mutations in *ELOVL4* cause autosomal dominant STGD3, an aggressive degeneration striking the macular region of the retina with juvenile onset and rapid progression (Bernstein et al., 2001; Edwards et al., 2001; Zhang et al., 2001; Maugeri et al., 2004; Bardak et al., 2016; Donato et al., 2018). Symptoms associated with STGD3 are limited to the retina, as STGD3 patients show no additional CNS disease or skin abnormalities (Bernstein et al., 2001; Edwards et al., 2001; Zhang et al., 2001). Importantly, STGD3-causing *ELOVL4* mutations result in a loss of function. STGD3 arises from several different mutations in exon 6 that result in premature termination of the protein and cause the loss of the C-terminal ER retention motif (Bernstein et al., 2001; Edwards et al., 2001; Zhang et al., 2001; Maugeri et al., 2004; Bardak et al., 2016). The 5 bp deletion STDG3 mutant form of ELOVL4 exerts a dominant-negative effect on the enzyme and leads to mislocalization of ELOVL4 away from the ER (Logan et al., 2013; Logan et al., 2014). Two additional STGD3-causing mutations in the *ELOVL4* promoter that suppress *ELOVL4* expression have been identified recently (Donato et al., 2018).

#### SCA34

Four different heterozygous mutations in the *ELOVL4* gene cause autosomal dominant spinocerebellar ataxia-34 (SCA34), a late-onset degenerative disease of the cerebellum that may present with or without erythrokeratodermia variabilis (EKV; red thickened skin) (Cadieux-Dion et al., 2014; Bourassa et al., 2015; Ozaki et al., 2015; Bourque et al., 2018). The gait ataxia and cerebellar degeneration that are characteristic of the disease appear in the second to sixth decade of life, with symptom onset varying according to the specific mutation. Other CNS symptoms also may be present, including dysarthria (difficulty speaking), abnormal eye movements, and abnormal tendon reflexes. Patients with SCA34 do not show any clinical retinal deficits. All of the known mutations that cause SCA34 are mutations in exon 4 that result in a single amino acid substitution and produce a full-length protein. The presence of neural deficits and degeneration in the brain appear in the absence of retinal symptoms in SCA34 patients, suggesting that SCA34-causing mutations in *ELOVL4* may preferentially affect synthesis of VLC-SFA, rather than VLC-PUFA. A recent study suggests that SCA34 patients with the p.T233M SCA34 mutation also may experience multi-system neurodegeneration beyond the cerebellum, neuropsychiatric disturbances, and dementia, in addition to the known motor deficits of SCA34 (Ozaki et al., 2019).

#### ELOVL4 Neuro-Ichthyotic Syndrome

Three different homozygous mutations in the *ELOVL4* gene cause a severe neuro-ichthyotic syndrome (Aldahmesh et al., 2011; Mir et al., 2014). The neural components of this syndrome include severe seizures, intellectual disability, spasticity, and neurodegeneration in the brain. These neurological impairments are accompanied by ichthyosis (a scaly thickening of the skin), developmental delay, and premature death. Mutations that cause this severe syndrome may be associated with truncation of the protein, whether affecting just the first few amino acids at the N-terminal (Mir et al., 2014), or the last C-terminal third of the protein (Aldahmesh et al., 2011), which includes the critical ER retention motif of ELOVL4. Interestingly, homozygous inheritance of STDG3 alleles in transgenic mice in which ELOVL4 has been rescued in the skin to prevent perinatal death due to dehydration, causes a syndrome characterized by severe seizures and early death about postnatal days 20–21 (Hopiavuori et al., 2018). No cases of homozygous inheritance of STGD3 or SCA34 *ELOVL4* alleles in humans have been reported to date.

### Neurophysiological Role of ELOVL4, Its Products, and Their Functions in the CNS and at the Synapse

Despite the obvious importance of ELOVL4 to the health and function of the CNS, the precise role and mechanisms of action of its main fatty acid products, VLC-PUFA and VLC-SFA, are incompletely understood. Recent evidence suggests that specific lipid species that contain each of these types of very-long acyl chains may have important roles, with VLC-PUFA serving as precursors of metabolites involved in homeostatic signals and VLC-SFA serving as modulators of synaptic transmission.

#### VLC-PUFA as Homeostatic Survival Signals

Recently, elegant studies of VLC-PUFA by the Bazan laboratory identified a new class of bioactive fatty acids they named “elovanoids” (Bhattacharjee et al., 2017; Jun et al., 2017; Bazan, 2018). These compounds, hydroxylated derivatives of 32:6n-3 and 34:6n-3 produced by a form of lipoxygenase, were described as neuroprotective in the retina., The primary products of ELOVL4 in the retina are VLC-PUFA that are incorporated into phosphatidylcholine and enriched in the disc membranes of the light-sensitive photoreceptor outer segments (Aveldano, 1987). Each morning, photoreceptors shed a portion of the discs at the distal tip of the outer segment, which are phagocytosed by the overlying retinal pigmented epithelium (RPE) and degraded. The VLC-PUFA in the shed outer segment membranes serve as the precursors for production of oxygenated elovanoid derivatives in RPE cells. Elovanoids then provide a neuroprotective feedback signal to enhance expression of pro-survival proteins by the photoreceptors to compensate for high levels of oxidative stress (Jun et al., 2017). In good agreement with these findings, elovanoids also have been shown to have protective effects in neurons subjected to oxygen and glucose deprivation or to induced excitotoxicity in culture, and in an animal model of ischemic stroke (Bhattacharjee et al., 2017). Together, these data revealed a novel pro-homeostatic and neuroprotective lipid-signaling mechanism that helps to sustain the integrity of neuronal cells. For more information on omega-3 and omega-6 unsaturated fatty acids, DHA, docosanoids, elovanoids and their biological functions, we refer to an excellent recent review from Bazan (2018).

#### VLC-SFA as Modulators of Synaptic Function

An emerging understanding suggests that VLC-SFA are important for normal synaptic function and that VLC-SFA deficiency arising from ELOVL4 mutations impairs synaptic transmission and causes synaptopathy. Recent studies performed using mice homozygous for the 5 bp deletion STDG3 mutation, which effectively renders ELOVL4 inactive, showed that VLC-SFA are important, novel modulators of presynaptic release kinetics (Hopiavuori et al., 2018). Of the potential roles that VLC-SFA may play in the CNS, we will consider two major areas below: as structural elements and as signaling molecule and signal modifiers. We also provide thoughts on future research directions to better understand the function of VLC-FA and their potential clinical relevance.

### Structural Role of VLC-SFA

Although much remains unknown about the role of ELOLV4 and its VLC-SFA products in neurophysiology, we suggest that VLC-SFA incorporated into sphingolipids in the synaptic vesicle membrane may potentially serve as membrane stabilizers, anchor points for proteins, and lipid raft-like components.

#### Potential Role for VLC-SFA in Membrane Fusion

One of the most precisely regulated membrane fusion events known occurs during neurotransmission in the synaptic terminal (Südhof, 2004; Südhof and Rothman, 2009). Membrane fusion is a fundamental step in neurotransmitter release when synaptic vesicles (SV) fuse with the plasma membrane at the active zone to release their neurotransmitter content into the synaptic cleft. Receptors on the postsynaptic side bind the transmitter and relay the signal to the next neuron. This process, which is the basis for information processing in the brain, has received considerable attention in human cognitive research and in neurodegenerative diseases such as Alzheimer’s and Parkinson’s diseases (Dickson, 1997; Selkoe, 2001, 2002; Petersen, 2004; Nguyen et al., 2006). The core machinery required for action potential evoked synchronized transmitter release and the molecular components needed for neurotransmission are now identified. This core complex contains three SNARE (soluble NSF attachment protein -SNAP- receptor) proteins (Sollner et al., 1993): the synaptic vesicle protein, synaptobrevin (also known as VAMP, Vesicle Associated Membrane Protein), and the target membrane bound syntaxin and SNAP-25 proteins. The coiled motifs of these three proteins form a four helical complex structure, thus bringing the membranes in close proximity (McNew et al., 2000; Melia et al., 2002). During action potentials, the fusion of the vesicular and active zone membranes is triggered by binding of calcium ions to synaptotagmin1, another vesicular protein, which binds preferentially to phospholipids (Perin et al., 1990; Fernandez-Chacon et al., 2001). Recently, an array of synaptic proteins (Munc13, Munc18, Rabphilin, and Complexin) that interact with the core complex and assist in the precise regulation of synaptic release have been discovered (Hata et al., 1993; Rizo and Sudhof, 2002; Deák et al., 2006b; Deák et al., 2009; Südhof and Rothman, 2009). Recently, the exact stoichiometry of these and other SV proteins and SV lipids was reported, and biophysical characterization of SV membranes was performed (Takamori et al., 2006; Hopiavuori et al., 2017; Hopiavuori et al., 2018). Regarding lipids, these authors showed that an average 40 nm diameter SV contains some 7000 phospholipid and 5-6000 cholesterol molecules. Together, the glycerophospholipids phosphatidylcholine, phosphatidylethanolamine, and phosphatidylserine comprise more than 90% of the phospholipid content, part of the rest being phosphatidylinositol, sphingomyelin, and hexosylceramide (Takamori et al., 2006). The most abundant VLC-SFA components of the SV membrane lipid were 28:0 and 30:0 incorporated into sphingolipids (Hopiavuori et al., 2018).

**FIGURE 7:**
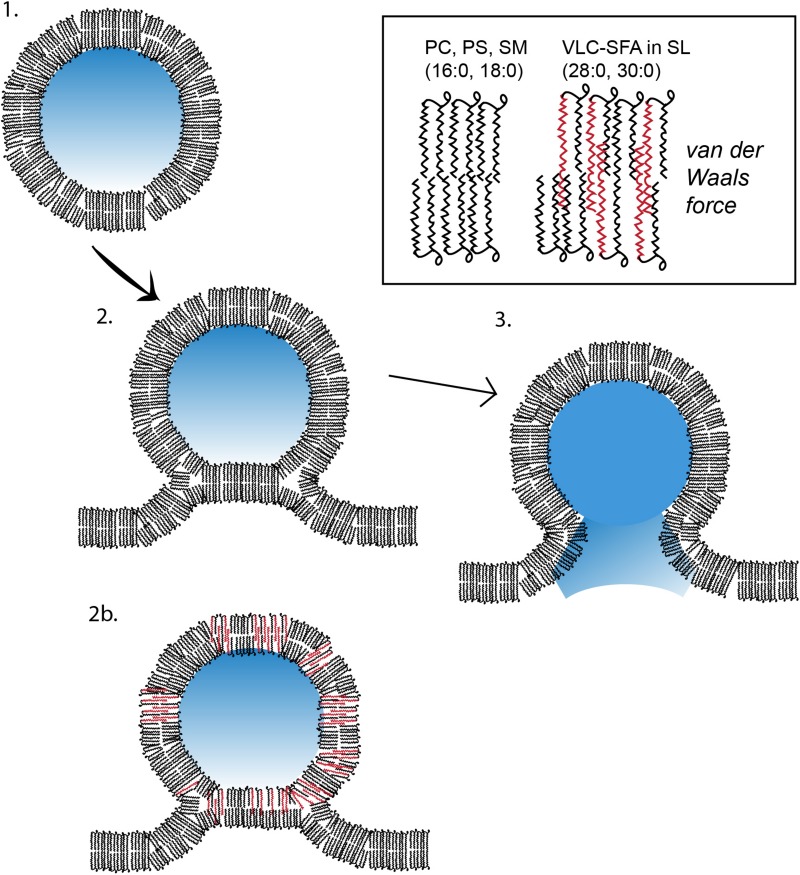
Model of membrane fusion between a VLC-SFA-rich synaptic vesicle and presynaptic active zone membrane. In this cartoon, a synaptic vesicle (SV) containing lipids with common fatty acids (phosphatidylcholine or phosphatidylserine molecules with palmitic, 16:0, and stearic acids, 18:0) is used as a model to illustrate the three stages of synaptic vesicular exocytosis, namely, (1) undocked vesicle; (2) docked and hemifused vesicle stage, with deformation tilt of the membranes and stalk formation; and (3) membrane fusion and contents release stage. In (2b), an SV is depicted with lipids containing, in addition, VLC-SFA (typically 28:0 or 30:0 fatty acid chains, see inset). We have shown that the normal presence of these fatty acids in the SV membrane renders it more stable than do the shorter LC fatty acids, thereby fusing and releasing at comparatively lower rates (Hopiavuori et al., 2018) (PS, phosphatidylserine; PC, phosphatidylcholine; SM, sphingomyelin; SL, sphingolipids; see text for more details).

During exocytosis, the membrane lipid bilayers merge in a stepwise process. First, the membrane leaflets exposed to the cytoplasm make contact each other and then, if forced with sufficient energy, will merge. Only after this process is initiated, and at least partially completed, can the other membrane leaflets – in the case of synaptic neurotransmission, the luminal leaflet of the synaptic vesicle and the extracellular leaflet of the plasma membrane – contact each other. Thus, an intermediate state – called hemifusion – exists when the outer leaflets of the vesicle and plasma membrane form a temporary new bilayer. After these outer leaflets merge, the vesicular lumen and the synaptic cleft remain separate. Next, after a fusion pore is formed, the release of neurotransmitter is initiated (Figure 7).

**FIGURE 8:**
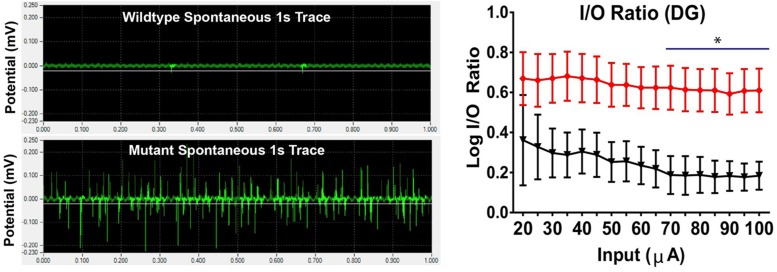
Seizure activity resulting from STGD3 deletion. **Left:** Spontaneous bursting activity in hippocampal slices from *S^+^Elovl4^mut/mut^* mice with knock-in of the 5 bp STGD3 mutation and skin-specific rescue of wildtype (WT) *ELOVL4* function to prevent perinatal lethality, and *S^+^Elovl4^wt/wt^* control mice expressing the WT *ELOVL4* gene under baseline conditions. Representative traces of field potentials from postnatal day 19 *S^+^Elovl4^wt/wt^* **(upper trace)** and *S^+^Elovl4^mut/mut^* mice **(lower trace)**. Each trace represents the first 1 s of a single channel chosen from a 10 min recording taken using a 64 channel multi-electrode array. The *S^+^Elovl4^mut/mut^* slice shows a dramatic increase in spontaneous neuronal activity compared to WT littermates. **Right:** Stepwise increased stimulations to perforant path synapses in dentate gyrus (DG) revealed a markedly enhanced input/output (I/O) ratio indicating a boost in synaptic strength in the main input pathway from the entorhinal cortex of *S^+^Elovl4^mut/mut^* mice (red) compared to *S^+^Elovl4^wt/wt^* control mice (statistics: two-way RM ANOVA, ^∗^*p* < 0.05 from 70 to 100 μA). Reproduced from Hopiavuori et al. (2018) with the permission under the Creative Commons Attribution 4.0 International License (link to the Creative Commons license; http://creativecommons.org/licenses/by/4.0/).

**FIGURE 9:**
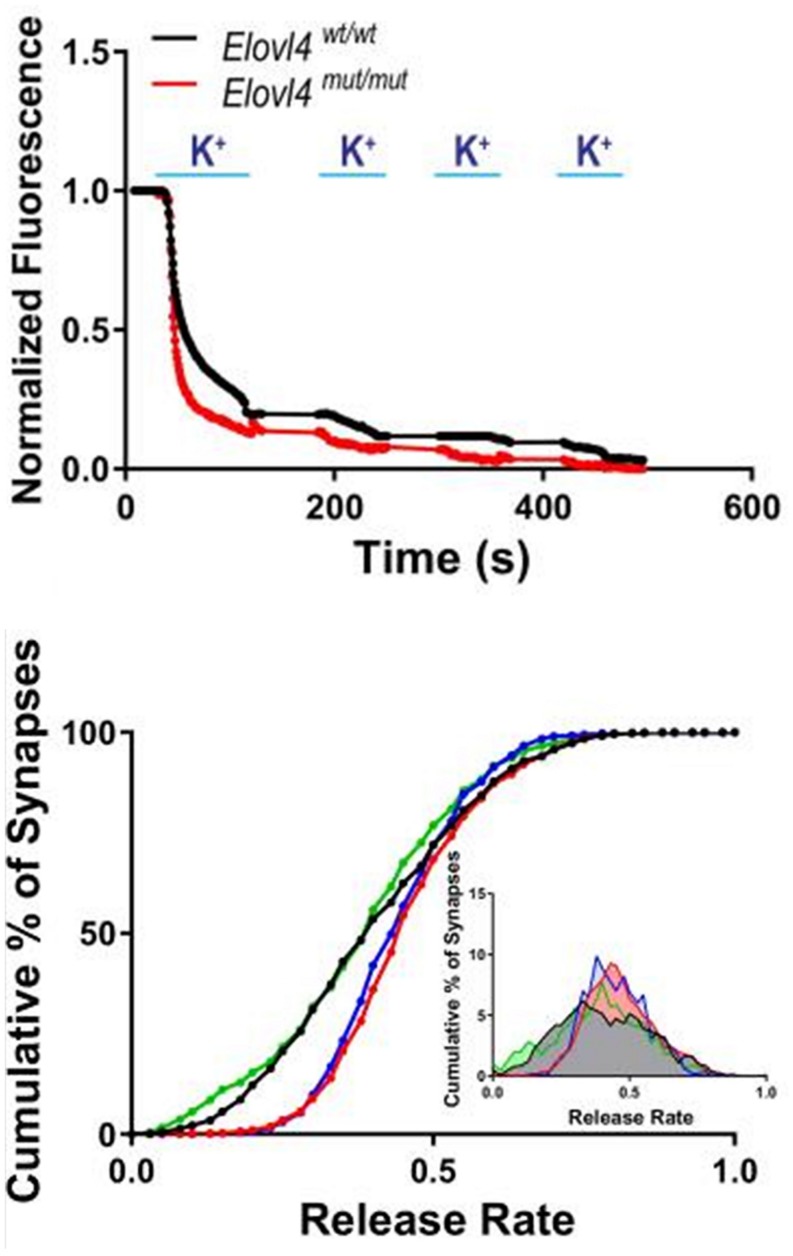
Accelerated synaptic vesicle release in *Elovl4* mutant neurons and its rescue by VLC-SFA. **Top panel:** Faster synaptic release in Elovl4^mut/mut^ hippocampal synapses (red) compared to WT control synapses (black), as detected with FM1-43 dye. Representative traces of average synaptic release activated by four rounds of high K^+^ depolarization. **Bottom panel:** Cumulative graph depicts the fraction of total releasable synaptic vesicle pool exocytosed after 15 s depolarization (WT = black line). Vesicle release kinetics was recorded by FM1-43 fluorescence from 900 to 1200 synapses. Note the robust right shift of the cumulative distribution for *Elovl4*^mut/mut^ synapses (red), which is rescued by supplementation with 28:0 and 30:0 SFAs (green) but not by 24:0 (blue). Inset: Frequency distribution of responses with slowest responding synapses on the left, and the fastest on the right on the curve. Reproduced from Hopiavuori et al. (2018) with permission under the Creative Commons Attribution 4.0 International License (link to the Creative Commons license; http://creativecommons.org/licenses/by/4.0/).

VLC-SFA potentially could change the structure of the lipid bilayer and its biophysical properties, thereby affecting the process of membrane fusion. The SV membrane contains 28:0 and 30:0 as components of sphingolipids (Hopiavuori et al., 2018). These very long saturated acyl chains are of sufficient length to traverse both leaflets of the SV lipid bilayer. We hypothesize that the hydrophobic van der Walls interactions between these very long chains stabilize the SV lipid bilayer, hindering the separation of the two leaflets during hemifusion and fusion pore formation. The enrichment of VLC-SFA in SV membranes, therefore, would increase the energy required to initiate vesicular fusion, stabilizing the membranes and reducing random spontaneous fusion events unrelated to action-potential mediated synaptic release. According to this model, acyl-acyl hydrophobic interactions across the lipid bilayer would increase the van der Waals forces within the bilayer, thereby stabilizing the membrane and inhibiting fusion with other membranes. Assuming that the energy source driving membrane fusion is constant, the vesicular release process would be slowed in SVs with high VLC-SFA content. The VLC-SFA in the vesicle membrane could thereby provide a biophysical resistance to the activation of SNARE complexes, which is essential for Ca^2+^-regulated exocytosis of synaptic vesicles (Deák et al., 2006a, 2009; Südhof and Rothman, 2009; Südhof and Rizo, 2011; Imig et al., 2014; Shen et al., 2015), slowing SV fusion. We suggest that this increased barrier may help to regulate the timing of vesicular release. *Elovl4* mutations that cause seizures (STDG3 and ELOVL4 neuro-ichthyotic syndrome) effectively eliminate the enzymatic function of ELOVL4 and, thus, lead to SVs that lack VLC-SFA. The absence of the van der Waals forces generated by the VLC-SFA within the SV lipid bilayer would therefore be expected to increase the probability of spontaneous release events by removing a barrier to fusion. In this case, the precise timing of synaptic release would be compromised, leading to bursting activity and seizures, as seen in the hippocampus of knock-in mice homozygous for the 5bp deletion STGD3 *ELOVL4* mutation (Figure 8; Hopiavuori et al., 2018). Consistent with this hypothesis, hippocampal neurons cultured from mice homozygous for the 5bp deletion STGD3 *ELOVL4* mutation show accelerated synaptic release kinetics (Hopiavuori et al., 2018). Critically, supplementation of a mixture of 28:0 and 30:0 VLC-SFA to these neurons via the culture medium restored normal synaptic release kinetics (Figure 9; Hopiavuori et al., 2018).

The length and saturation of VLC-SFA also may enable them to sterically inhibit protein-protein interactions in the vesicular release machinery. However, interactions between VLC-SFA and the vesicle fusion machinery remain unknown at present. VLC-SFA interactions are also likely to keep the membrane bilayers highly ordered and stable, thus hindering the effects of synaptobrevin juxta- and trans-membrane regions in disturbing outer leaflet lipid ordering and changing curvature of membrane (Tarafdar et al., 2015). It is also plausible for charged phospholipids (e.g., sphingolipids) containing VLC-SFA to interact with the release proteins via a strongly charged segment in synaptobrevin/VAMP located next to its transmembrane domain outside of the SV (Quetglas et al., 2000).

What other functional consequences might result from the presence of VLC-SFA in the SV membrane? It is well established that the SNARE protein syntaxin1A forms clusters in the active zone (Takamori et al., 2006; Khuong et al., 2013). Interestingly, the syntaxin1A clusters are dispersed in the absence of PI(3,4,5)P_3_ at *Drosophila* larval neuromuscular junction (Khuong et al., 2013). Could VLC-SFA containing sphingolipids, similarly, cluster synaptobrevin/VAMPs, the vesicular SNARE binding partners of syntaxins in the SV membrane? Although an intriguing possibility, it is unlikely that islands of VLC-SFA in the membrane would serve as anchoring points for vesicular SNAREs, as the expected effect of VLC-SFA deficiency would be to disorganize the VAMPs, which would lead to slower release. Exactly the opposite of the observed effect of VLC-SFA deficiency in the mouse model (Hopiavuori et al., 2018). The presence of VLC-SFA could impose some ordering of proteins at the fusion site beyond the SNARE proteins, though, as many accessory or modulatory vesicular proteins have transmembrane domains (e.g., Synaptotagmins) or membrane anchoring lipid modifications. Interestingly, the role of VLC-SFA in synaptic release is markedly different from that described for cholesterol, which facilitates synchronized evoked transmitter release (Wasser et al., 2007; Linetti et al., 2010; Teixeira et al., 2012). Miniature synaptic potentials arise from single SV release in the absence of action potentials (Katz and Miledi, 1979; Sara et al., 2005). Therefore, these events are useful direct indicators of membrane fusion dynamics; future studies could address the effect of VLC-SFA on miniature release.

Alternatively, the effect of VLC-SFA anchoring points should be considered for vesicular events that do not complete fusion and intermixing of vesicular membrane with the plasma membrane. Neurophysiologists call such partial fusion events as “Kiss and run” (Fesce et al., 1994; Stevens and Williams, 2000) when the fusion pore is reversibly closed and the vesicle escapes intact without merging fully into the active zone membrane (Gandhi and Stevens, 2003; Südhof and Rizo, 2011). It remains to be tested whether VLC-SFA alter the preference for the fusion mode from total exocytosis to kiss-and-run events.

Another intriguing question is whether VLC-SFA are components of lipid rafts. Lipid rafts are specific microdomains of the plasma membrane, which differ in their composition from the rest of the plasma membrane (Jacobson et al., 2007). Formation of rafts requires cholesterol, and these cholesterol- and sphingolipid-rich plasma membrane microdomains play important roles in compartmentalization of cellular functions. Lipid rafts, which range in size from 10 to 200 nm in diameter, comprise various fractions of the plasma membrane depending on the cell type, and provide a mechanism for ordering clusters of proteins within them (Hao et al., 2001). The detergent-insoluble lipid rafts contain proteins that are modified posttranslationally by acylation or glycosylphosphatidylinositol (GPI) modification. Some as yet unidentified interactions between VLC-SFA and other components of the lipid rafts could increase the stability of the raft domains. Alternatively, the long acyl chain length of VLC-SFA are suited perfectly to the increased width of the lipid bilayer in the rafts.

VLC-PUFA also are potential modulators of synaptic function. Due to their polyunsaturated structure and large size, VLC-PUFA could regulate membrane fluidity and curvature by disrupting phospholipid packing of the lipid bilayer of synaptic membranes, similarly, to DHA and other PUFA (Antonny et al., 2015; Lauwers et al., 2016). The retina provides one example for such a role of VLC-PUFA: depletion of VLC-PUFA from mouse photoreceptors by conditional knockout of *Elovl4* reduced photoreceptor synaptic vesicle diameter compared to synaptic vesicle diameter in wildtype photoreceptors (Bennett et al., 2014b). This change in diameter potentially could affect vesicle fusion or transmitter content. Consistent with this notion, the amplitude of signals in the electroretinogram that reflect transmission of rod photoreceptor signals to the inner retina, also was reduced in the conditional *Elovl4* knockout mice (Bennett et al., 2014a). For more detailed insight on VLC-FA function in the retina and vision, we refer to our colleague’s most recent review (Hopiavuori et al., 2019).

### Potential Signaling Functions of VLC-SFA and VLC-PUFA

**FIGURE 10:**
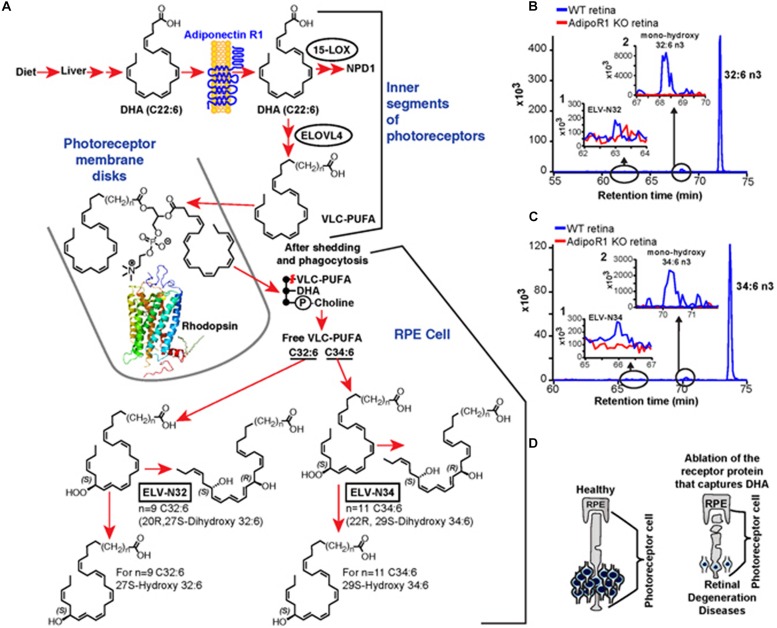
Elovanoid signaling derivatives of VLC-PUFA. Genetic ablation of adiponectin receptor 1 leads to depletion of VLC-PUFAs and its derivatives in retina. **(A)** Dietary DHA, or that derived from dietary 18:3n3, is supplied by the liver and captured by adiponectin receptor 1 (AdipoR1), followed by elongation in the inner segment of PRC by ELOVL4 to VLC-PUFA and incorporation into phosphatidylcholine molecular species, which also contains DHA. During daily PRC outer segment renewal, these phosphatidylcholine molecular species interact with rhodopsin and, after shedding and phagocytosis, become part of RPE cells. UOS or other disruptors of homeostasis trigger the release of VLC-PUFAs. 32:6n3 and 34:6n3 are depicted generating hydroperoxy forms, and then ELV-N32 or ELV-N34, respectively. **(B)** The pool size of free 32:6n3 in retinas of AdipoR1 knockout (KO) mice (red) is decreased as compared with that in wild type (WT) (blue). Insert (1) shows ELV-N32 in KO (red) and WT (blue); insert (2) shows mono-hydroxy 32:6n3, the stable derivative of the hydroperoxy precursor of ELV-N32, in WT (blue) and lack of detectable signal in the KO (red). **(C)** Similarly, the pool size of free 34:6n3 in retinas of AdipoR1 KO mice (red) is decreased as compared with that in WT (blue). Insert (1) shows ELV-N32 in KO (red) and WT (blue); insert (2) shows mono-hydroxy 34:6n3, the stable derivative of the hydroperoxy precursor of ELV-N34, in WT (blue) and lack of detectable signal in the KO (red). **(D)** RPE cells sustain PRC functional integrity (left); right, the ablation of AdipoR1 switches off DHA availability, and PRC degeneration ensues. Reproduced from Jun et al. (2017) with permission under the Creative Commons Attribution 4.0 International License (link to the Creative Commons license; http://creativecommons.org/licenses/by/4.0/).

VLC-SFA and VLC-PUFA potentially also might tune synaptic transmission through inter-neuronal interactions. As discussed above, elegant studies by the Bazan laboratory identified a new class of bioactive oxygenated VLC-PUFA derivatives they named elovanoids, including mono-hydroxy 32:6n3, the stable derivative of the hydroperoxy precursor of elovanoid-N32, which has neuroprotective activity in the retina (Figure 10; Jun et al., 2017). Alternatively, VLC-SFA or VLC-PUFA could potentially serve as an initial substrate for novel messenger pathways to provide either intracellular or trans-synaptic signals that would modulate synaptic function. For example, endocannabinoids provide retrograde signals to modulate presynaptic function via receptor-mediated signaling (Wilson and Nicoll, 2002; Fan and Yazulla, 2004). Another example to be considered is intracellular signaling via the PLC-PIP2-IP3/DAG pathway.

Proper intracellular membrane trafficking depends on the phosphatidylinositol-phosphate (PIP) system. Although PIPs are a minor fraction of the membrane lipids, they are essential for proper targeting of vesicular traffic and for synaptic endocytosis (Di Paolo et al., 2004; Zoncu et al., 2007). The cytoplasmic side of cellular membranes acquires an asymmetrical PIP profile through an elaborate regulatory system of selective PI kinases, including PIPKgamma (Wenk et al., 2001), PIPK4 (Guo et al., 2003), and phosphatases. Importantly, PIPs also interact with various ion channels. For instance, modulation of transient receptor potential (TRP) channels by phosphoinositides, especially PIP_2_, is well established (Rohacs and Nilius, 2007; Rohacs, 2015). Whether VLC-FA can exert similar effects on integral membrane proteins such as ion channels is a fascinating untested question.

### Open Questions, Future Directions

#### Trafficking of VLC-SFA-Containing Membranes From Endoplasmic Reticulum (ER) to SV

Although there is abundant information on ER trafficking from yeast and other single cell organisms (Novick and Schekman, 1979; Balch et al., 1984; Kaiser and Schekman, 1990), the details of SV formation are incompletely understood. Because the enzymes that elongate fatty acids, including ELOVL4, localize to the ER, questions arise about how lipids containing the VLC-SFA get selectively incorporated into synaptic vesicles, during the step of vesicle budding and fusion that precedes the release of vesicles from the ER. Before vesicle budding, v-SNARE and transmembrane cargo proteins from the ER membrane collect into the ER budding zone and recruit the proximal and distal coat assembly, leading to a vesicle bound by a neck to the ER membrane (Bonifacino and Glick, 2004). The cargo then becomes concentrated, membrane curvature increases, and vesicle scission occurs. It is tempting to speculate that there may be interaction between lipids containing VLC-SFA and specific transmembrane cargo proteins of the ER. It may also be envisaged that specific proteins of the proximal coat assembly, which regulate the coordinated sorting of vesicles, play a role in selectively trapping the lipids with VLC-SFA into vesicle populations destined to become SVs.

Targeting of vesicles to different organelles requires highly coordinated modification of the PIPs in their membranes. For instance, PIPK4 phosphorylates PIP_2_ in SV (Guo et al., 2003). Interestingly, contact sites between virtually every organelle and the ER have been identified, and the functional importance of these small, specialized membrane domains is increasingly recognized. Recent developments have highlighted the role of PI-4-P gradients as critical determinants of the non-vesicular transport of various lipids from the ER to other organelles such as the Golgi or plasma membrane (Balla, 2018).

In addition to the ER at the soma, a well-developed smooth ER (SER) in neuronal axons has been known for nearly half a century (Droz et al., 1975). The axonal ER has important biosynthetic functions including synthesis of axonal proteins and nerve ending components (Luarte et al., 2018). An interesting possibility is that the lipid composition of SVs originating from the somatic ER might be modified at the axonal SER during their axonal transport. One can also hypothesize that VLC-SFA may be synthesized there and added to sphingosine (by one of the Cer synthases) or exchanged with an ordinary (16:0) FA-containing sphingolipid at the SER by lipid remodeling. It is clear that most ELOVL4 is localized to the somatic SER, but the possibility that a small fraction of ELOVL4 might be present in axonal SER outposts cannot be ruled out.

### Further Clinical Implications of VLC-FA

#### Epilepsy and Ketogenic Diet

Epilepsy patients are prescribed a ketogenic diet – a fat-rich diet that is low in sugars – when all anti-convulsive drugs fail to reduce seizures (Cervenka et al., 2013; Sharma and Tripathi, 2013). The clinical effectiveness of the ketogenic diet (including medium-chain triglyceride diet) has been confirmed in a number of clinical trials carried out mainly on children (Liu, 2008; Kayyali et al., 2014; Kossoff et al., 2018), but its application may be limited by the number of early (gastrointestinal distress, acidosis, hypoglycaemia, dehydration and lethargy), and late adverse effects (hyperuricemia, hyperlipidaemia, kidney stones, easy bruising, and decreases in height and weight) (Ballaban-Gil et al., 1998; Kang et al., 2004; Ulamek-Koziol et al., 2016). With strict monitoring and adapting the diet to the needs of the individual patient according to recently updated guidelines (Kossoff et al., 2018), it is possible to avoid most of these complications. The mechanism by which the ketogenic diet helps to control seizures remains unknown (Kayyali et al., 2014). Assessing changes of VLC-FA levels in the CNS associated with ketogenic diet and seizure disorders could provide valuable insight into the pathophysiology of epilepsy and help to develop more effective therapies.

#### Dementia and Alzheimer’s Disease

There is tremendous interest in nutritional interventions to prevent cognitive impairment in the elderly, including the potential of fatty acids to prevent or slow cognitive impairment (Bazan et al., 2002; Sinn et al., 2010; Calon, 2011; Jiao et al., 2014; Burckhardt et al., 2016; Rangel-Huerta and Gil, 2018). Change in cell membrane lipid composition has been suggested to be associated with cognitive impairment in Alzheimer’s disease (Drolle et al., 2017; Diaz et al., 2018; Penke et al., 2018). Moreover, association between beta-amyloid (Aβ), a main pathological hallmark of Alzheimer’s, and arachidonic acid in erythrocyte membrane was found to be specific to Apolipoprotein E ε4 non-carrier patients (Hooper et al., 2017). Treatments with DHA or other PUFAs have not provided significant protection from cognitive decline in the elderly thus far (Carrie et al., 2009; Dacks et al., 2013; Jiao et al., 2014; Phillips et al., 2015; Nishihira et al., 2016; Zhang et al., 2016), however, assessment of treatments including lipids with longer fatty acids than DHA have started only recently. Ongoing massive clinical trials, like the Cognitive Ageing, Nutrition, and Neurogenesis (CANN) trial, are presently testing potential benefits of combined dietary intervention with long-chain n-3 PUFA and flavonoids on cognition in older adults with mild cognitive impairment or subjective memory impairment (Irvine et al., 2018). The studies summarized in this review pose a number of stimulating questions and open future research directions to explore the potential applicability of VLC-FA to the treatment of specific human brain and retina diseases, like cognitive impairment, epileptic seizures and macular degeneration.

## In Summary

Recent studies reveal novel functional roles for ELOVL4 and its VLC-FA products in the CNS, including retina and brain, in health and disease. VLC-PUFA play vital functions in the CNS as precursors of compounds that serve newly recognized roles in homeostatic signaling and regulation of neuronal survival. Most recently it was shown that VLC-SFA have essential functions in synaptic transmission, with disruption of VLC-SFA synthesis leading to seizures and neurodegeneration both in patients lacking ELOVL4 and in mouse models of ELOVL4 mutations. A better understanding of VLC-SFA and VLC-PUFA metabolism and their functions in the CNS holds great promise for development of new therapeutic avenues for treatment of seizures and neurodegenerative diseases.

## Author Contributions

FD, RA, JF, and DS contributed to the writing and editing of the manuscript.

## Conflict of Interest

RA has a United States Patent (No. 8,021,874) entitled “Very Long Chain Polyunsaturated Fatty Acids, Methods of Production, and Uses.” The remaining authors declare that the research was conducted in the absence of any commercial or financial relationships that could be construed as a potential conflict of interest.
